# Changes in retinal metabolic profiles associated with form deprivation myopia development in guinea pigs

**DOI:** 10.1038/s41598-017-03075-3

**Published:** 2017-06-05

**Authors:** Jinglei Yang, Peter S. Reinach, Sen Zhang, Miaozhen Pan, Wenfeng Sun, Bo Liu, Fen Li, Xiaoqing Li, Aihua Zhao, Tianlu Chen, Wei Jia, Jia Qu, Xiangtian Zhou

**Affiliations:** 1 School of Ophthalmology and Optometry and Eye Hospital, Wenzhou Medical University, Wenzhou, Zhejiang China; 2 State Key Laboratory Cultivation Base and Key Laboratory of Vision Science, Ministry of Health, China, and Zhejiang Provincial Key Laboratory of Ophthalmology and Optometry, Wenzhou, Zhejiang China; 3 Shanghai Key Laboratory of Diabetes Mellitus and Center for Translational Medicine, Shanghai Jiao Tong University Affiliated Sixth People’s Hospital, Shanghai, 200233 China

## Abstract

Retinal metabolic changes have been suggested to be associated with myopia development. However, little is known about either their identity or time dependent behavior during this sight compromising process. To address these questions, gas chromatography time-of-flight mass spectrometry (GC-TOF/MS) was applied to compare guinea pig retinal metabolite levels in form deprivation (FD) eyes at 3 days and 2 weeks post FD with normal control (NC) eyes. Orthogonal partial least squares (OPLS) models discriminated between time dependent retinal metabolic profiles in the presence and absence of FD. Myopia severity was associated with more metabolic pattern differences in the FD than in the NC eyes. After 3 days of FD, 11 metabolite levels changed and after 2 weeks the number of differences increased to 16. Five metabolites continuously decreased during two weeks of FD. Two-way ANOVA of the changes identified by OPLS indicates that 15 out of the 22 metabolites differences were significant. Taken together, these results suggest that myopia progression is associated with an inverse relationship between increases in glucose accumulation and lipid level decreases in form-deprived guinea pig eyes. Such changes indicate that metabolomic studies are an informative approach to identify time dependent retinal metabolic alterations associated with this disease.

## Introduction

Myopia incidence is reaching epidemic proportions in some populations, and may have a progressively negative impact on quality of life^1^. In severe cases, this disease can give rise to sight-compromising complications and even blindness arising from glaucoma and retinal detachment^2^. Environmental factors play a role in the modulation of myopia progression as there is an association between the time school-age children spent outdoors and disease suppression^3^. This association was validated in myopia animal models, in which increases in light exposure suppressed myopia progression^4, 5^. Genetic factors also contribute since a large number of loci have been reported for either common or high myopia^6^, but for the vast majority of these myopias the exact genetic variants are still unknown. Epidemiological and clinical research findings also suggest that myopia etiology involves interactions between various genetic and environmental factors^7^.

Emmetropization and the homeostatic control of eye growth is strongly guided by visual input signals^8^. However, little is known about how visual signals initiate myopia related pathway alterations and direct axial elongation. Changes in gene expression of a number of growth factors have been reported to be associated with myopia, including hepatocyte growth factor^9^, transforming growth factor beta 1^10, 11^ and basic fibroblast growth factor^12^. Dysfunction of neurotransmitters including those in the dopaminergic^13^, gamma-aminobutyric acid-ergic (GABAergic)^14, 15^ and cholinergic^16^ systems are reported to be associated with this condition. These results imply that metabolic alterations also occur in the retina. However, little is known about what time dependent metabolite changes occur during myopia development.

Metabolomic studies elucidate the metabolic signatures underlying disease processes by providing a direct readout of the physical conditions and metabolic status of tissues and organs^17^. This approach has aided in gaining insight into the pathogenic mechanisms underlying various cancers, diabetes and psychosis as well as eye disease^18, 19^. Nuclear magnetic resonance identified biomarker characteristics of patients afflicted with lens-induced uveitis or chronic uveitis^20^. Global and targeted mass spectrometry vitreous humor analysis suggested that an arginine to proline upregulation would be a therapeutic option in diabetic retinopathy^21^. Similarly, examination of metabolic pathways involving tyrosine and urea generation may provide insight into the pathophysiology of age-related macular degeneration^22^. A comparison of vitreous humor metabolite levels in choroidal detachment and posterior vitreoretinopathy demonstrated that reductions in arachidonic acid biosynthetic products are characteristic of these pathologies^23^. Notably, a very recent report used capillary electrophoresis in combination with mass spectrometry to identify 20 different metabolite changes in human aqueous humor content as a function of myopia severity^24^. Their findings suggest that oxidative stress could contribute to myopia progression since arginine and citrulline were at higher concentrations in high myopia samples. Another relevant difference between high and low myopia was that increases in didehydro-retinoic acid precede both proteoglycan synthesis upregulation and axial length extension.

We used GC-TOF/MS to probe for time-dependent differences in retinal metabolic patterns that occur during form deprivation myopia (FDM) in guinea pigs. Our findings suggest that myopia development is associated with declines in lipid biosynthesis, tricarboxylic acid (TCA) cycle turnover as well as increases in glucose accumulation.

## Materials and Methods

### Animals

Two-week-old pigmented guinea pigs (*Caviaporcellus*, English short-hair stock, tricolor strain) were obtained from the animal laboratory center of Zhejiang University in Hangzhou, China. All animals were reared under a 12-h light/12-h dark cycle in the animal facilities with the room temperature maintained at 25 °C. Guinea pigs had free access to standard food and water with fresh vegetables provided twice daily. All procedures are consistent with the ARVO Statement for the Use of Animals in Ophthalmic and Vision Research. The Animal Care and Ethics Committee at Wenzhou Medical University, Wenzhou, China approved the animal research.

### Establishment of axial myopia

**Figure 1:**
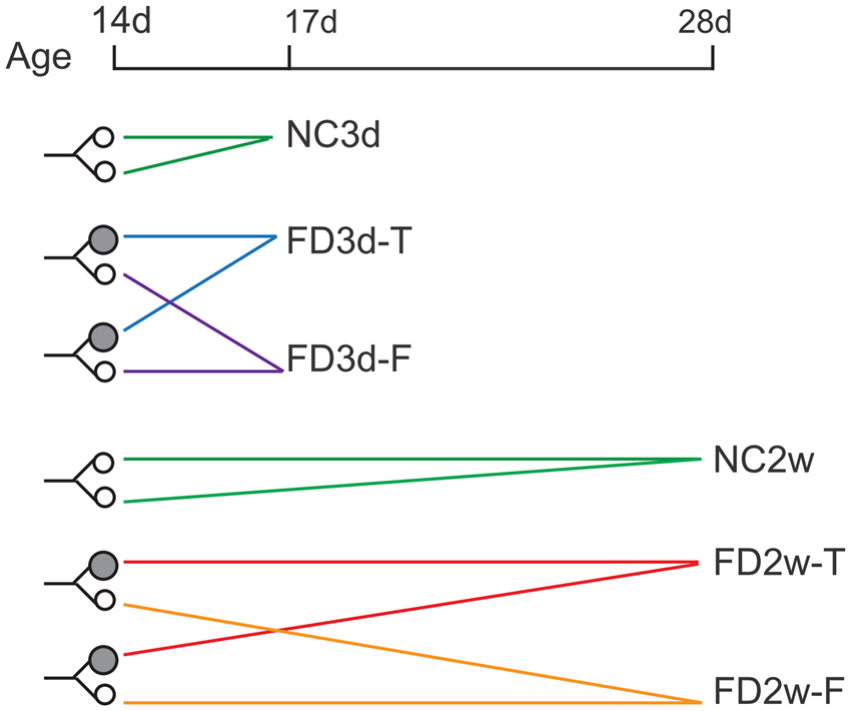
Group assignment paradigm. Guinea pigs were randomly assigned to FD groups and NC groups at an age of 14 days. Animals in the FD groups were monocularly deprived, whereas those in the NC groups did not receive any treatment. A filled circle indicates an FD eye, and an open circle indicates an unpatched F eye. Twenty-four animals in the FD and 10 in NC groups were sacrificed 3 days later (i.e., 17 days of age), and the eyes were designated as FD3d and NC3d, respectively. Two FD eyes from 2 animals in the FD3d group were pooled together and designated as FD3d-T, and the two F eyes were pooled and named FD3d-F. Twenty-four animals in the FD and 12 in NC group were sacrificed at the age of 28 days and designated as NC2w and FD2w. Two FD eyes from 2 animals in the FD2w group were pooled together and designated as FD2w-T, and the two F eyes were pooled and designated as FD2w-F.

Pigmented guinea pigs were randomly assigned to form deprivation (FD) groups and normal control (NC) groups. FD was established using a monocularly deprived facemask made of latex balloons (Suzhou, China) to cover only the right eye^25^. The uncovered left eye is designated as the fellow (F) eye. The nose, mouth and ears were also not covered. Facemask integrity was examined and re-positioned three times weekly and immediately changed with a new mask of suitable size when necessary. Twenty-four animals were stopped from FD after 3 days and sacrificed along with 10 age-matched controls. Another 24 animals were stopped from FD after 2 weeks and sacrificed with 12 age-matched controls. Figure 1 illustrates the experimental paradigm. The eyes were labeled as follows: FD, the form deprived eyes including FD3d-T(obtained 3 days post FD) and FD2w-T(obtained 2 weeks post FD); F, the contralateral of FD eyes including FD3d-F and FD2w-F; NC: eyes from control animals including NC3d and NC2w.

## Biometric measurements

Refraction of the eyes was measured in the pupil vertical meridian using an eccentric infrared photorefractor^26^ provided by Professor Frank Schaeffel (University of Tübingen, Germany). There are some modifications in the software based on refractive status of guinea pigs. Guinea pigs were amenable to handling and could be readily aligned towards the camera without general anesthesia, which provided clear visible pupil images in the video frame. Illumination of the examination room was dimmed to approximately 5 lux. Three refractive measurements were recorded, and the mean was used for statistical analysis.

Axial components of the eye were measured in unanesthetized animals using A-scan ultrasonography (11 MHz, AVISO Echograph Class I-Type Bat; Quantel Medical, Clermont-Ferrand, France)^27^ on the same day following refraction measurements. The cornea was topically anesthetized with a drop of 0.5% proparacaine hydrochloride (Alcon). The recorded parameters included anterior chamber depth (ACD), lens thickness (LT), vitreous chamber depth (VCD), and axial length (AL; distance from anterior surface of cornea to vitreal facing surface of retina). Each parameter was measured at least 8 times, and the mean was used for analysis. The facemask of FD animals was removed before and replaced immediately after each measurement.

### Retinal metabolic profiling

Guinea pigs were immediately sacrificed after biometric measurements via cervical dislocation at the end of an experimental period. Each eye was enucleated and placed on an inverted ice-cold culture dish for dissection of the cornea, lens and the optic nerve head, leaving an eyecup. The entire retina with all its layers was carefully isolated from the eyecup. Each sample contained two retinas to obtain sufficient metabolites: left and right eyes from each animal were pooled in the NC groups, and two patched and unpatched eyes from two different animals were individually pooled to form the FD and fellow eye groups (Fig. 1). Retinas were weighed and frozen in liquid nitrogen followed by storage at −80 °C prior to experimentation.

Metabolites were analyzed using chemical derivatization according to procedures described for brain samples with minor modifications^28^. Briefly, each sample was homogenized in 250 µl of a solvent mixture (chloroform: methanol: water = 1:2:1, v/v/v) for 2 min using a Mini-beadbeater-24 (Biospec, Bartlesville, OK, USA) and stored for 20 min at −20 °C. The samples were centrifuged at 12,000 × g for 10 min. A total of 150 µl of aqueous supernatant was transferred to a GC vial containing two internal standards, L-2-chlorophenylalanine (10 µl, 0.3 mg/mL) and heptadecanoic acid (10 µl, 1.0 mg/ml). The precipitate was re-homogenized for 30 sec at 4 °C after the addition of 250 µl of methanol. The samples were centrifuged at 12,000 × g for 10 min and another 150 µl aliquot of supernatant was added to the mixture in the GC vial and vacuum dried. The residue was derivatized using a two-step procedure. First, 80 µl of methoxyamine (15 mg/ml in pyridine) were added to the vial and maintained at 30 °C for 90 min, followed by the addition of 80 µl of derivatization solution (N,O-bis(trimethylsilyl) trifluoroacetamide (BSTFA): trimethylchlorosilane (TMCS) = 99:1, Sigma Aldrich Co., China) and maintained at 70 °C for 60 min.

Data were collected and analyzed as described^29^. Each 1-µl aliquot of the derivatized solution was injected in splitless mode into an Agilent 6890N gas chromatograph coupled with a Pegasus HT time-of-flight mass spectrometer (Leco Corporation, St. Joseph, MI). Samples were successively analyzed in a control sequence of pre- and post-treatment. Electron impact ionization (70 eV) at full-scan mode (m/z 30–600) was used with an acquisition rate of 20 spectra/s in the TOF/MS setting. The MS files acquired from GC-TOFMS analysis were exported in NetCDF format using ChromaTOF software (v3.30, Leco Co., CA). CDF files were extracted using custom scripts (revised MATLAB toolbox hierarchical multivariate curve resolution) using MATLAB 7.0 (The MathWorks, Inc.) for data pretreatment procedures, such as baseline correction, background noise suppression, smoothing, alignment, time-window splitting, and multivariate curve resolution (based on a multivariate curve resolution algorithm). The resulting three-dimensional data set included sample information, peak retention time and peak intensities. Internal standards and any known artificial peaks, such as peaks caused by noise, column bleed and BSTFA derivatization procedure, were removed from the data set. Compound identification was performed by comparing the mass fragments with NIST 05 Standard mass spectral databases in NIST MS search 2.0 (NIST, Gaithersburg, MD) software. If a similarity was identified of greater than 70%, it was verified by comparison with available reference compounds. Data for the identified compounds were mean-centered and unit variance-scaled during chemometric data analysis for multivariate statistical analysis using the SIMCA-p 11.5 Software package (Umetrics, Umeå, Sweden).

### Statistical Analysis

#### Unidimensional statistical analyses

All measured ocular parameters in the right and left eyes were compared using paired *t*-test. One-way ANOVA with Bonferroni correction was used to compare biometric results between FD and age-matched NC group. A p value of less than 0.05 was considered as significant. This part of statistical analysis was performed using SPSS (Version 16.0). Abundance of each metabolite between FD and NC as well as FD and F groups were compared using two-way ANOVA with time and treatment (i.e. FD) as factors by MATLAB. The adjusted p value was then calculated using the procedure introduced by Storey^30^.

#### Multidimensional statistical analyses

Principal component analysis (PCA), partial least squares discriminate analysis (PLS-DA) and orthogonal partial least squares (OPLS) were performed to discriminate between groups. The following statistical measures, based on the models, are discussed in detail throughout this article. R^2^X is the modeled variation in the X matrix; R^2^Y is the modeled variation in the Y matrix; and Q^2^Y is the predicted variation in the Y variable or matrix, based on seven-fold cross-validation. R^2^Xcum, R^2^Ycum and Q^2^cum are the cumulative sums of R^2^X and Q^2^Y. The range of these parameters is 0 to 1, where 1 indicates a perfect fit. Random permutation of the Y observations while keeping the X-matrix intact was used to measure whether and by how much the data were overfitting^31^. The default 7-round cross-validation in the SIMCA-p software package was applied with one-seventh of the samples being excluded from the mathematical model in each round to guard against overfitting. It was possible to obtain a number of variables responsible for the difference in the metabolic profiles of the groups based on the variable importance on a projection (VIP) threshold (VIP > 1.0) of an appropriate 7-fold cross-validated OPLS model.

## Results

### Retinal metabolic profile deviation induced by form deprivation

**Figure 2:**
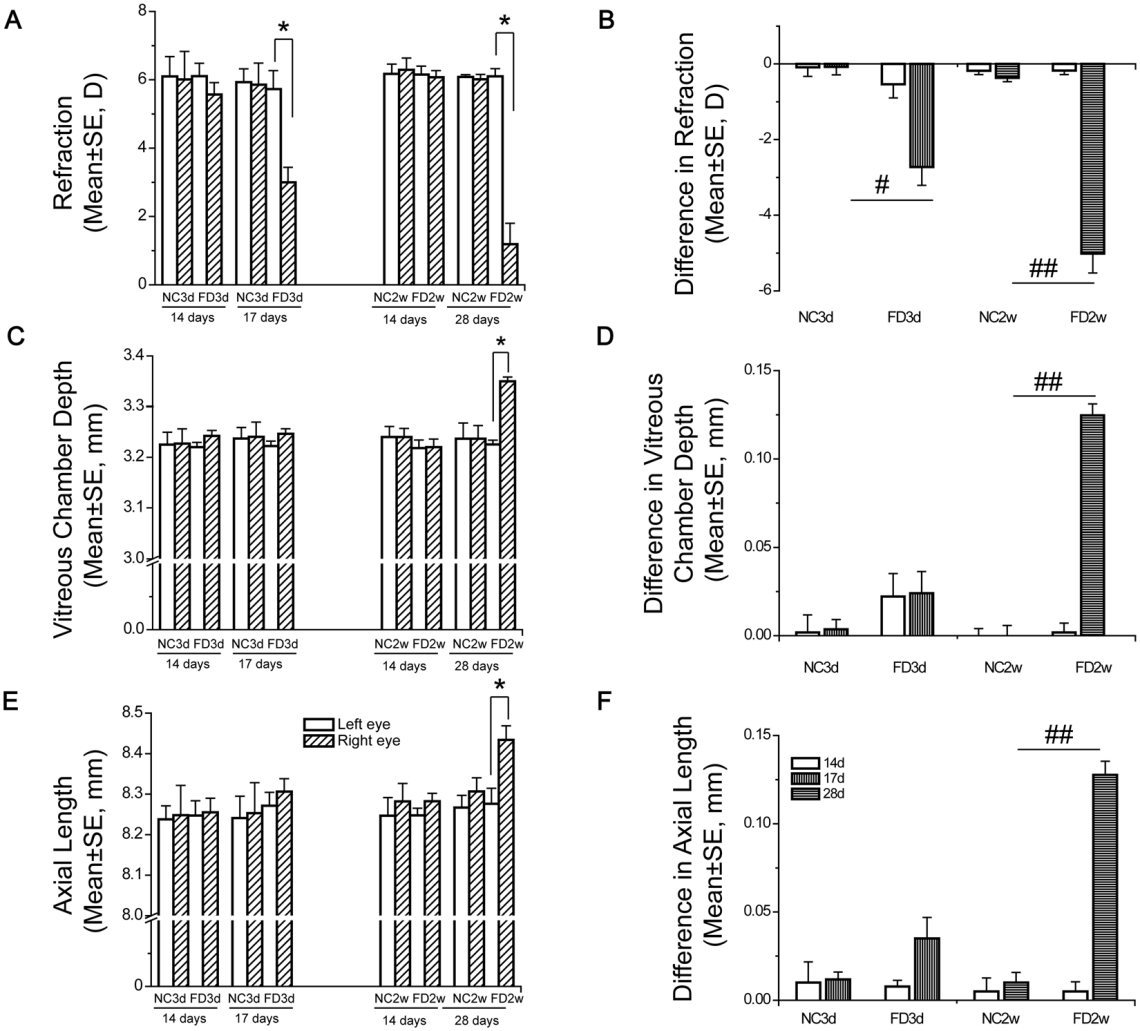
FD induced axial myopia after 2 weeks. Panels (A), C and E show refraction, vitreous chamber depth and axial length of animals in NC3d and FD3d at age of 14 and 17 days, and those of animals in NC2w and FD2w at age of 14 and 28 days. Panels (B,D and F) show differences in refraction, vitreous chamber depth and axial length before and after 3 days of FD for animals in NC3d and FD3d, and differences before and after 14 days of FD for animals in NC2w and FD2w. Sample number: 10 in NC3d, 24 in FD3d, 12 in NC2w, 24 in FD2w. Data are shown as mean ± standard error of the mean(SE). The asterisk in (**A**,**C** and **E**) signifies a significant difference between right and left eyes within a group (p < 0.05). Hashtag symbol in (**B**,**D** and **F**) designates a significant difference between FD group and respective control group (^#^p < 0.05; ^##^p < 0.01).

There were no significant differences between the ocular parameters of left and right eyes of two-week-old animals prior to initiating FD, which included refraction, VCD and AL (Fig. 2, *P* > 0.05, paired *t*-test). Form-deprived animals were monocularly deprived for 3 days and 2 weeks (FD3d and FD2w, respectively) and compared with age-matched NC animals (NC3d and NC2w, respectively) to evaluate time-dependent metabolome changes. There were no differences in refraction or axial components between the right and left eyes in the NC3d and NC2w groups, which is consistent with our previous report^26^. The form-deprived groups developed myopia of −2.73 ± 2.35D and −5.01 ± 2.53D after 3 days and 2 weeks, respectively. Previous reports show that FD induced a myopic shift of 2.21 ± 2.11 D after 2 weeks^25^ and 4.06 D after 11 days^32^. The degree of myopia seems to be larger herein, perhaps because in previous studies FD was imposed in 3 week old guinea pigs while we used 2 week old animals when refractive error development is more sensitive to visual distortion. There was also a trend towards an increase in VCD and AL elongation after FD, but only the interocular difference in AL after 2 weeks of FD was significant. No such changes in any of the optical parameters occurred in the F eyes.

Retinal metabolic profiles were obtained in form-deprived eyes (N = 12 from 24 animals) and F eyes from FD3d and FD2w (N = 12 from 24 animals) and control eyes from NC3d (N = 5 from 10 animals) and NC2w (N = 6 from 12 animals). On the average, 600 peaks were obtained using gas chromatography on each sample, and 101 peaks were identified according to their retention time and ions in the mass spectrometer. Their identity was verified by comparisons with available known standards in our library as well as those in the public NIST library. This expression pattern of 101 metabolites was designated as a retinal metabolic profile. Metabolic profiles in the form-deprived retinas were compared with those obtained from the F eyes and age-matched NC counterpart.

**Figure 3:**
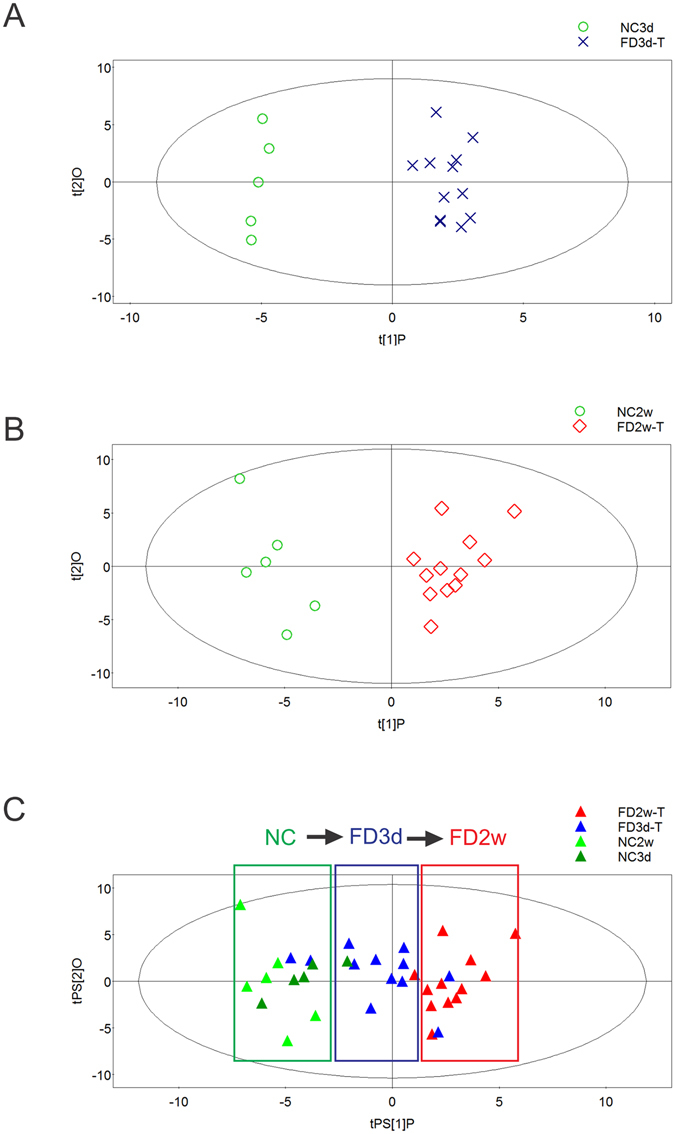
FD induced retinal metabolic profile shifts. Based on the content of 101 identified retinal metabolites, metabolome changes induced by 3 days and 2 weeks of FD were different from age-matched NC3d (**A**) and NC2w (**B**) using OPLS models. Samples from FD3d-T localized between FD2w-T and NC2w in the t predicted plot using NC3d and FD3d-T as a prediction set of the OPLS model discriminating FD2w-T and NC2w (**C**), which indicates an association between FDM development and metabolic profile changes. t[1]P: score calculated with predictive components; t[2]O: score calculated with orthogonal components. tPS[1]P: score calculated with predictive components of samples in prediction set (PS); tPS[2]O: score calculated with orthogonal components of samples in PS.

We performed PCA to compare the metabolic profiles of FD3d-T, FD3d-F and NC3d eyes. On the PC1 and PC2 axes, all samples clustered together (data not shown), indicating the similarity of the metabolic profiles among each treatment condition. However, an OPLS scores plot using 1 predictive component and 1 orthogonal component (R^2^Xcum = 0.276, R^2^Ycum = 0.972, Q^2^cum = 0.715) revealed intrinsic differences between FD3d-T and NC3d (Fig. 3A), indicating there are changes in retinal metabolites associated with FD. This is also the case for time point 28 days. Two weeks after experiments began, unsupervised PCA plot reveals little differences between FD2w-T, FD2w-F and NC2w. An OPLS score plot using 1 predictive component and 1 orthogonal component (R^2^Xcum = 0.415, R^2^Ycum = 0.912, Q^2^cum = 0.644) revealed a difference between FD2w-T and NC2w (Fig. 3B). In the validation, Q^2^cum intercepted the Y-axis at −0.10 in the 999 random permutations test, and the controlled model was considered well protected from overfitting (Supplementary Fig S1). These findings strongly suggest that there are intrinsic differences between FD2w-F and NC2w which may be associated with FD.

As metabolic profile differences after 2 weeks of FD seem to be related to myopia progression, the OPLS model was used to differentiate between FD2w-T and NC2w profiles and to predict group variables of FD3d-T and NC3d. The sensitivity and specificity of this OPLS model as a classifier was 75% (3 of 12 FD-3T were predicted as normal) and 100% (none of NC3d was predicted as myopia), respectively. Samples from the NC3d groups localized around NC2w samples in the t predicted plot, which indicated no changes in their metabolic profiles between 17 and 28 days. Samples from FD3d-T localized between NC3d/NC2w and FD2w-T samples (Fig. 3C). Obvious shifts occurred in the metabolic profiles from NC to FD3d and FD2w eyes that were associated with the severity of myopia.

### Metabolic pattern shifts during FD

To resolve the time dependence of metabolic shifts occurring during FD lasting up to three weeks, levels of each metabolite in form deprived eyes were compared with those in F and NC eyes at two time points, i.e. 3 days and 2 weeks post FD.

Differences in 11 metabolites were significant in FD3d versus NC3d and 16 were in FD2w versus NC2w (Table 1). To delineate the time dependence of the different pattern changes, they were separated into three groups: 1) 6 that only changed up to 3 days, i.e., early responders; 2) 5 changed continuously throughout the entire two week FD period; 3) 11 that changed just between 3 and 14 days, namely, late responders.

**Table 1:** Differential metabolite profiles during myopia development.

Compound	FD3d versus NC3d				FD2w versus NC2w				p value
	FC		VIP		FC		VIP		
	T/C	T/F	T vs C	F VS T	T/C	T/F	T vs C	F VS T	T vs C
Mannose	2.69	0.50	***2.29***	***1.21***	1.95	1.26	0.70	0.49	0.060
Urea	0.69	0.85	***2.22***	***1.40***	0.86	1.00	***1.00***	0.00	**0.012**
Glucose	1.37	0.82	***1.93***	***1.18***	1.31	0.98	***1.22***	0.19	**0.012**
Arabinose	0.45	0.85	***1.44***	***1.45***	0.92	0.88	0.31	***1.04***	0.148
Tyrosine	0.86	0.72	***1.00***	***1.63***	0.91	0.87	0.52	***1.13***	0.096
Glutamic acid	0.90	0.85	***1.03***	***1.31***	0.91	0.99	0.82	0.12	0.112
**Threonine**	0.79	0.78	***2.00***	***1.48***	0.86	0.87	0.95	***1.17***	**0.013**
**Valine**	0.87	0.80	***2.05***	***1.81***	0.85	0.83	***1.16***	***1.86***	**0.011**
**Isoleucine**	0.85	0.78	***1.98***	***1.90***	0.81	0.81	***1.38***	***2.02***	**0.010**
**Malic acid**	0.85	0.93	***1.38***	0.62	0.78	0.88	***1.38***	***1.15***	**0.012**
**Alanine**	0.94	0.84	***1.04***	***1.67***	0.87	0.85	***1.15***	***1.72***	**0.032**
Arachidic acid (20:0)	0.93	1.04	0.26	0.14	0.26	0.66	***2.20***	***1.94***	**0.010**
Octadecenoic acid (18:1)	0.96	0.93	0.45	0.52	0.73	0.94	***2.02***	***1.29***	**0.010**
Octadecanoic acid (18:0)	1.03	0.94	0.43	0.52	0.82	0.92	***1.84***	***1.38***	**0.041**
Arachidonic acid (20:4)	0.96	0.96	0.54	0.38	0.80	0.94	***1.78***	***1.94***	**0.014**
Cholesterol	0.98	0.93	0.43	0.67	0.80	0.92	***1.72***	***1.39***	**0.012**
Ethanolamine	1.08	0.95	0.38	0.29	0.79	0.85	***1.66***	***2.13***	0.179
Hexadecanoic acid (16:0)	1.04	0.90	0.66	0.79	0.80	0.93	***1.61***	***1.12***	**0.042**
Tetradecanoic acid (14:0)	0.91	0.93	0.90	0.57	0.71	0.87	***1.59***	***1.25***	**0.013**
Octadecadienoic acid (18:2)	0.90	0.90	0.57	0.52	0.66	1.19	***1.43***	***1.23***	**0.031**
2-Ketoglutaric acid	1.51	0.69	0.84	***1.28***	2.61	0.68	***1.09***	***1.33***	0.047
GABA	0.98	0.89	0.33	***1.21***	0.87	0.93	***1.08***	***1.10***	0.090

FC: fold change, abundance of individual metabolites in the former versus the latter group. T: FD eyes; F: fellow eyes; C: normal control eyes. VIP: variable importance in the OPLS model discriminating two groups. P values are from two-way ANOVA assessing main effect of treatment type (with or without FD), adjusted by FDR method with q value set at 0.05. Compound changes with VIP > 1.0 and P < 0.05(shown in italic bold) were regarded as significant. The five listed boldface compounds were significantly different in both FD3d versus NC3d and FD2w versus NC2w. Except for them, the first 6 compounds in the table were significantly different in FD3d versus NC3d and the bottom 11were significantly different in FD2w versus NC2w.

In the early responder group, mannose, urea, glucose, arabinose, tyrosine and glutamic acid were significantly different in FD3d versus NC3d, suggesting that their changes are either contributing to myopia progression or occur in response to FD. As for carbohydrate metabolites, mannose and glucose levels in this group rose in the FD eyes compared with those in the NC retinas, while arabinose and glutamic acid levels fell. Levels of the amino acid tyrosine fell concomitantly with its metabolic end product, urea.

Threonine, valine, isoleucine, alanine and malic acid levels, were significantly different in both FD3d versus NC3d and FD2w versus NC2w, and continually decreased during FD. These amino acid declines could reflect increases in protein biosynthesis or conversion to metabolites in metabolic energy conserving pathways. On the other hand, malic acid declined suggesting that TCA cycle activity fell, which makes the latter alternative less likely.

Among the 11 compounds in the late responder group, 7 of them are involved in unsaturated fatty acid biosynthesis (www.kegg.jp, map01040) and declined (Arachidic acid, octadecenoic acid, octadecanoic acid, arachidonic acid (ARA), hexadecanoic acid, tetradecanoic acid, octadecadienoic acid).

Except for multidimentional statistical analysis, two-way ANOVA was performed to identify whether the abundance of each retinal metabolite changed with respect to time and/or FD. Metabolites in FD eyes were compared with those in NC as well as F eyes followed by using the post-hoc FDR method. No interaction was found between time and FD as all p values were statistically non-significant after FDR correction. Fifteen metabolites among the 22 differences identified by OPLS were also different between FD and NC eyes (p values after FDR correction are provided in Table 1). None of the metabolite levels were different between FD and F eyes.

## Discussion

It is a daunting task to identify metabolic signatures in myopia that may provide insight into the identification of potential drug targets to reverse this disease process. We applied the form deprivation myopia animal model to address this issue. We observed time-dependent refractive error development and corresponding VCD and AL growth in guinea pigs after form deprivation. The differences in refraction, VCD and AL between goggled and contralateral eyes are statistically significant after 2 weeks of FD. At 3 days post FD, only the difference in refraction is significant whereas the VCD and AL differences did not attain significance even though they became somewhat larger. We expect that these biometric parameter differences would reach significance if the sample size was larger.

A total of 101 different metabolites were identified, including carbohydrates, fatty acids and amino acids in guinea pig retinas. Figure 3D clearly shows time-dependent deviation of metabolic patterns after FD from 3 days to 2 weeks away from the NC condition suggesting an association between FD and changes in energy conserving metabolism as well as unsaturated fatty acid biosynthesis.

One suggestion that such changes could be related to myopia progression in humans is that this process was slowed in school-aged children who were fed a diet containing large amounts of animal protein (10% of energy)^33^. The present study implicates an energy conserving metabolic component to myopic progression because glucose accumulated in the form-deprived eyes suggesting a decline in aerobic glycolysis. Even though glucose accumulated, the TCA cycle activity change appears to be complex. On the other hand, the α-ketoglutarate level increased whereas the malate level fell suggesting a possible inhibitory effect in the pathway linking together these two cycle substrates. Seven intermediates involved in lipid metabolism decreased during FD relative to NC eyes suggesting fatty acid biosynthesis inhibition. Therefore, our results suggest that myopia progression is associated with an inverse relationship between increases in glucose accumulation and reductions in lipid biosynthesis.

Seven out of the 16 metabolites that either changed continuously or after a 3 day delay are constituents of a lipid metabolic pathway (www.kegg.jp, map01040). The declines in ARA levels and fatty acid precursors and derivatives in the form-deprived eyes suggest that myopia development is associated with ARA biosynthesis downregulation. Alternatively, these declines may reflect increases in the formation of metabolic products other than those identified in this study that are also derived from ARA. Prostaglandin levels were not measured in this study, but their levels may also decline because they are derived from ARA. A decline in the prostaglandin PGF2α may promote myopia progression because an injection of PGF2α into the vitreous suppressed FDM development in chicks^34^. The relevance of the declines in valine, iso-leucine and cholesterol to myopia development in form-deprived and F eyes is also an open question. Arachidic acid with a VIP of 2.2 is also a lipid, but it is not possible to interpret the significance of its 70% decline because of a lack of reports on its function. Nevertheless, this very substantial reduction coupled with the preponderant contribution of changes in lipid metabolic pathway constituents to myopia progression suggest that future studies using lipidomics are likely to improve our understanding of myopia development.

Notably, some of the metabolic shifts in the FD eyes were also observed in the unpatched F eyes, but their levels either increased or fell less than those in the corresponding FD eye. Siegwart *et al*. found changes in mRNA levels in form-deprived eyes and F eyes that occurred in the same direction, which are similar to the parallel declines in metabolic content between the form-deprived and F eyes in the present study^35^. These authors did not address why the F eyes underwent similar changes to those occurring in the FD eyes even though the F eyes do not develop myopia. There are two possibilities to account for this similarity in shifts between the FD and F eyes. Some aspect of our experimental procedure may have induced an unknown systemic effect and caused some of the metabolic pattern changes expressed in the FD eyes to also occur in the F eyes. Another possibility is that the platform sensitivity was inadequate to detect other changes that were unique to the FD eyes. It is conceivable that liquid chromatography may provide insight into this question because gas chromatography has limited sensitivity to detect polar metabolites although the derivatization method used herein facilitates the evaporation of some of these components^36^. Other monitoring platforms, such as configurations including liquid chromatography mass spectrometer (LC-MS), may have adequate sensitivity to identify differential metabolite levels between form deprived and NC eyes. One study used this technique in parallel with capillary electrophoresis and MS to characterize water soluble metabolic profile patterns in the aqueous humor from patients with high and low myopia^24^. In the study by Barbas-Bernardos *et al*., CE–MS detected over 40 metabolites in a small volume of aqueous humor composed of a vast number of amino acids and derivatives.

We detected most of the known amino acids in the retina and found that tyrosine, threonine, valine, isoleucine and GABA decreased during myopia progression. In contrast to the study quantifying myopic aqueous humor content^24^, arginine and citrulline were larger in high myopia in their study while we found that they were invariant. Dodecanedioic acid decreased in higher myopia in their study while we identified decreases in longer carbon-chain lipids including arachidic acid, octadecenoic acid, octadecanoic acid, arachidonic acid, hexadecanoic acid, tetradecanoic acid, octadecadienoic acid. The differences between our results and theirs may be attributable to firstly the fact that their analysis was made on aqueous humor samples whereas ours used retina, and secondly their platform is more sensitive to water soluble metabolites.

Collectively, this study demonstrated that metabolomics is a viable platform for the delineation of time-dependent retinal metabolic changes that occur during FD-induced myopia in guinea pigs. The declines in lipid content warrant further consideration using LC-MS to identify possible drug targets to suppress myopia development.

## Limitations

1. As myopia development is a time dependent process, we evaluated metabolic pattern changes as a function of time in our FD experimental model. Multidimensional OPLS identified 5 different metabolites undergoing changes whereas two-way ANOVA found that none of the metabolite changes were significant with respect to time as all p values were statistically non-significant after FDR correction. To attempt to resolve this discrepancy, we intend to measure metabolic profiles at more time-points.
2. Each FD, F and NC sample contained two retinas. Even though combining samples increases metabolite levels, this method renders variance between animals undetectable. Nevertheless, we were able to resolve differences between groups. In our preliminary experiment, we resolved approximately 450 peaks from one retina, 600 from two and 650 from three retinas. We pooled two retinas in one sample to resolve as many metabolites as possible and at the same time minimize animal use.
3. Identification of unknown metabolites is a major hurtle in metabolomics. Among mass-based platforms, GC/MS is a relatively mature method because of its acceptable reproducibility of sample measurements and adequate sensitivity to resolve between 200 to 500 entities in a biological sample. Usually only about 100 of them can be identified while the remaining are left unknown^37^. Our protocol for peak identification is very rigorous. Peaks are annotated when: i) they appear in more than 80% of the retinal samples; ii) their retention times coincide with standard mass peaks in our library including 1000 common annotated endogenous metabolites; iii) mass peak shapes coincide with those in the public NIST library.
4. Some peaks were unidentified, and some of these metabolites may be associated with myopia onset. This limitation may contribute to explain why the metabolic profiles of FD and F eyes are so similar to one another. Admittedly it is possible that there are other unidentified metabolite changes that we are missing that prevent us from more fully describing metabolic pattern changes occurring during myopia progression. We are now making a concerted effort to deal with this problem.

## Electronic supplementary material

Supplementary Statistical Analysis Descriptions
