# Supplementary material for: Changes in retinal metabolic profiles associated with form deprivation myopia development in guinea pigs
Source: Sci Rep. 2017 Jun 5;7:2777. doi: 10.1038/s41598-017-03075-3 (PMC5459838; doi:10.1038/s41598-017-03075-3)
Supplement: Supplementary file 1 — Supplementary Statistical Analysis Descriptions [file 41598_2017_3075_MOESM1_ESM.pdf]

Changes in retinal metabolic profiles associated with form deprivation myopia development in guinea pigs

Jinglei Yang[1,2], Peter S. Reinach[1,2], Sen Zhang[1,2], Miaozhen Pan[1,2], Wenfeng Sun[1,2], Bo Liu[1,2], Fen Li[1,2], Xiaoqing Li[1,2], Aihua Zhao[3], Tianlu Chen[3], Wei Jia[3], Jia Qu[1,2], Xiangtian Zhou[1,2]

1 School of Ophthalmology and Optometry and Eye Hospital, Wenzhou Medical University, Wenzhou, Zhejiang, China

2 State Key Laboratory Cultivation Base and Key Laboratory of Vision Science, Ministry of Health, China, and Zhejiang Provincial Key Laboratory of Ophthalmology and Optometry, Wenzhou, Zhejiang, China

3 Shanghai Key Laboratory of Diabetes Mellitus and Center for Translational Medicine, Shanghai Jiao Tong University Affiliated Sixth People's Hospital, Shanghai 200233, China

Correspondence:

Xiangtian Zhou, School of Ophthalmology and Optometry and Eye Hospital, Wenzhou Medical University, 270 Xueyuan Road, Wenzhou, Zhejiang, China 325027; zxt-dr@wz.zj.cn.

Jia Qu, School of Ophthalmology and Optometry and Eye Hospital, Wenzhou Medical University, 270 Xueyuan Road, Wenzhou, Zhejiang, China 325027; jqu@wz.zj.cn.

1. Multidimentional statistical methods

1.1 Principal component analysis (PCA)

PCA shows the correlation structure of data matrix X, approximating it by a matrix product of lower dimension (TP'), called the principal components plus a matrix of residuals (E).

$$X = 1 * \overline{X} + TP' + E$$

This geometric representation corresponds to fitting a line, plane or hyper plane to the data in multidimensional space with the variables represented as axes. The scaling of the variables specifies the length of the axes in this space. T is a matrix of scores that summarizes the X-variables, and P is a matrix of loadings showing the influence of the variables. E is a matrix of residuals; the deviations between the original values and the projections.

R2X is the fraction of the Sum of Squares (SS) of all the X's explained by the current component. R2X(cum) is the cumulative SS of all the X's explained by all extracted components.

1.2 Partial least squares (PLS)

PLS finds the linear (or polynomial) relationship between a matrix Y (dependent variables)

and a matrix X (predictor variables) expressed as:

$$Y = f(X) + E$$

The PLS model meeting these objectives can be expressed as:

$$X = 1 * \overline{X} + TP' + E$$

$$Y = 1 * \overline{Y} + UC' + F$$

$$U = T + H$$

T: Matrix of scores that summarizes the X variables
W: Matrix of weights expressing the correlation between X and U (Y)
U: Matrix of scores that summarizes the Y variables
C: Matrix of weights expressing the correlation between Y and T (X)
E, F, H: Matrices of residuals

In the PLS algorithm, there are additional loadings, W, called weights. They express the correlation between U and X and are used to calculate T.

This modeling geometrically corresponds to fitting a line, plane or hyper plane to both the X and Y data represented as points in a multidimensional space, with the objective of well approximating the original data tables X and Y, and maximizing the covariance between the observation positions on the hyper planes.

R2Y and R2X are the fraction of the Sum of Squares (SS) of all the Y's and X's explained by the current component. R2Y(cum) and R2X(cum) is the cumulative SS of all the Y's and X's explained by all extracted components.

1.3 Orthogonal PLS (OPLS)

Because of the complexity, uncertainty and ambiguity of biological samples, data redundancy removal is usually applied before the implementation of PLS. OPLS is one of the widely used and effective supervised pattern recognition methods. This method in advance takes correlation analysis of each of its columns (each variable) with the guide vector Y on the basis of PLS. If the variable has small correlation with Y, or the classification contribution of its orthogonal variable under the guidance of Y is considered to be ignored, the variable will not be used in modeling. Variables having large correlation with Y will be retained. Therefore, OPLS separates the PLS components into two groups:

Components that are related to Y are called predictive;

Components that are orthogonal to Y are called orthogonal.

With one Y, there is one predictive component and several orthogonal (no relation to Y) or pseudo orthogonals (when missing values are present in X) with very little relation to Y.

2. Validation

The order of Y is randomly permuted a number of times (1000 in our study) and separate models are fitted to all the permuted Y's extracting as many components as was done with the original matrix Y.

SS: Sum of Squares

RSS: Residual Sum of Squares

PRESS: PREdicted Sum of Squares

R2=1-RSS/SS.

Q2 =1.0 - PRESS/SS

R2X and R2Y denote the fraction of the Sum of Squares (SS) of all the Y's and X's explained by all the components. Q2 denotes the fraction of the total variation of the X's that can be predicted by a component, as estimated by cross-validation.

The SIMCA-P software displays the plot of the correlation coefficient between the original Y and the permuted Y (x axis) versus the cumulative R2Y and Q2 of all the components (y axis), and draws the regression line. The intercept is a measure of the over fit.

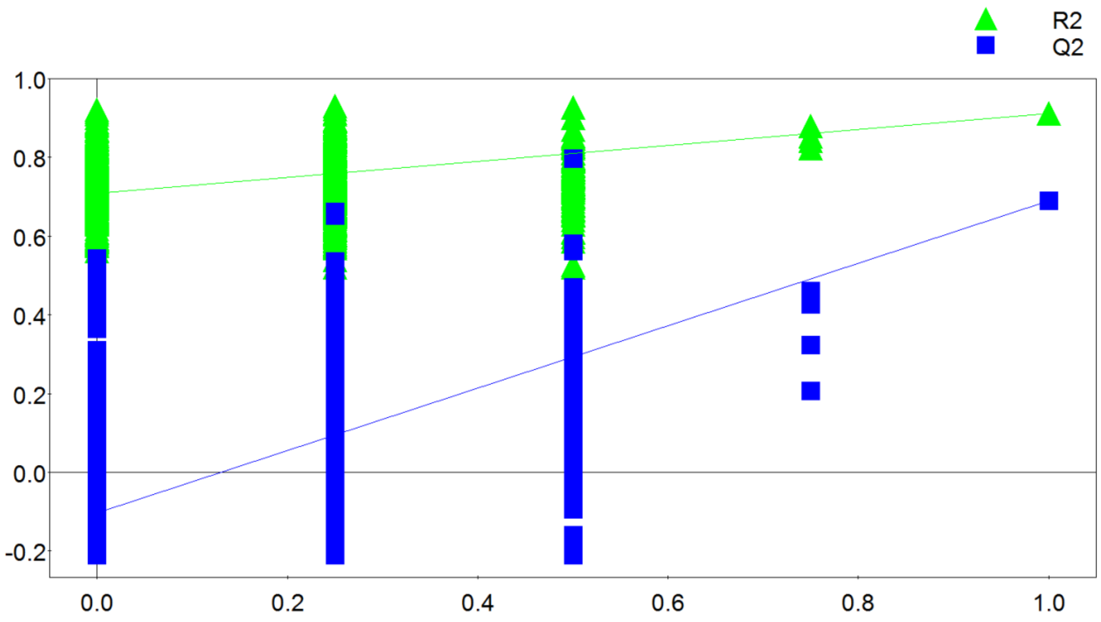

Figure S1 Validation of PLS model comparing metabolite changes between FD2w-T and NC2w.

The PLS model is considered well protected from overfitting since Q2cum intercepted the Y-axis at -0.10 in the 999 random permutations test.
